# Effectiveness of life skills training based on self-care on mental health and quality of life of married Afghan women in Iran

**DOI:** 10.1186/s12905-022-01875-y

**Published:** 2022-07-15

**Authors:** Fatemeh Abbasi Shovaz, Hassan Zareei Mahmoodabadi, Maryam Salehzadeh

**Affiliations:** Department of Psychology and Educational Sciences, Yazd University, Yazd, Iran

**Keywords:** Life skills, Self-care, Mental health, Quality of life, Afghan women

## Abstract

**Background:**

One of the major issues that immigrants, especially Afghan women face, is their self-care disability. This will cause problems in their mental health and quality of life. The aim of this study was to evaluate the effectiveness of life skills training based on self-care on mental health and quality of life of married Afghan women living in Taft.

**Methods:**

This quasi-experimental study was conducted using a pretest posttest design with control group. Statistical population was all married Afghan women living in Taft (Yazd province) of whom 60 women were selected using purposive sampling and were randomly allocated to two groups of 30 as experimental and control groups. The experimental group received 8 sessions of life skills training based on self-care. General Health Questionnaire (GHQ) and Quality of Life of the World Health Organization Questionnaire (WHOQ-BREF) were used to collect data. Multivariate analysis of covariance (MANCOVA) was used to analyze the data.

**Results:**

Results showed that following the intervention, the mean scores of quality of life (*p* < 0.0001) and mental health (*p* < 0.019) in the experimental group increased in the posttest compared to the pretest and the effect of intervention was significant.

**Conclusion:**

Results suggested that providing a training opportunity for Afghan women to learn life skills based on self-care, enables them to realize their strengths and weaknesses and improve their quality of life and mental health.

## Background

Life skills are a set of abilities for adaptive and positive behavior that enable individuals to accept their social responsibilities without harming themselves and effectively encounter daily challenges and problems [1].

Life skills are considered an essential source to develop mental, social, emotional, cognitive, and behavioral skills and flexibility to face daily challenges and have productive participation in society [2]. Therefore, it is essential to obtain knowledge and skills and change people's attitude to determine how programs are designed to increase life skills and to ensure individuals' access to the content and knowledge of the training program. Given the importance of life skills to prevent and promote mental health, teaching these skills seems essential [3]. All life skill development programs have the same goal, which is to help people overcome challenges and turn into healthy people [4].

Today, the concept of quality of life is a basic health indicator. Quality of life and the resulting mental health are integral to individuals. The physical and social settings where people live affect their social experiences, mental health, development and adaptation [5]. Quality of life is a fundamental concept in psychology and a powerful force to guide, maintain and promote health and wellness in different communities and cultures. The World Health Organization (WHO) defines four dimensions for the quality of life including physical health, mental health, social relations and the living environment [6, 7].

Mental health is a basic need to improve the quality of human life and is related to the inner empowering characteristics or inner interests of individuals [8]. It is a major public health concern as it makes a major contribution to pathogenesis of diseases around the world [9]. Mental health is the foundation of well-being and performance for individuals and the society, and women's health is important for both their own health and well-being of their children and family [10]. Women are more likely affected by mental disorders compared to men, the most common of which are anxiety and depressive disorders [11].

The life skills training program was developed to promote mental health and prevent social harms. The basic premise of designing this program is to help individuals learn life skills in addition to acquiring professional and occupational skills which will assist them to act effectively about themselves, others and society as a whole [12]. Self-care is the first step to health which teaches individuals how to protect themselves. About 65 to 85% of the care that leads to mental health and better quality of life is the result of self-care; i.e. activities that the family or ourselves do to maintain or promote mental health and quality of life. Life skills training based on self-care enables individuals to turn their knowledge, values and attitudes into actual abilities; i.e. one knows what to do and how to do it [13].

In recent decades, immigration has led to a wider range of studies on the mental health of immigrants. Besides, immigration itself causes psychiatric disorders, and factors such as poor economic status, dialectal and cultural differences, separation from relatives and the reasons that lead to immigration play a major role in exacerbating these disorders. Immigrants in host countries are often deprived of significant mental health services [14]. One of the major issues that immigrants, especially Afghan women face, is their self-care disability[6]. Therefore, life skills training based on self-care in Afghan women allows them to live more comfortably in the social environment with awareness of preventive skills and care, and to influence their comfort, performance, and health through learning self-care skills. In order to promote Afghan women's quality of life, increasing their social and interpersonal skills help them to decide consciously and communicate effectively, develop their coping skills and enjoy a healthier life. Higher health means more physical, mental and social comfort which affect the whole family and help correct others' behaviors. In case of receiving life skills training based on self-care as a principle in personal and social life, it will prevent the prevalence of harmful behaviors. In addition, life skills are integral to human life as acquired skills. Those with poor life skills fail to use their capacities well and are unable to meet their need compared to those who have these skills [15].

The results of previous studies revealed that the life skills training are effective in improving the quality of life, reducing perceived psychological stress and promoting women's health [16, 17]. In a study conducted on the quality of life of immigrants in China, it was found that life skills training before immigration with an emphasis on developing efficient coping skills improve quality of life and mental health [18].

In this study, since Afghan women have been away from their homeland for a long time and have suffered a lot of pressure, and on the other hand, they had to play their maternal and caring role well, it was essential to teach them life skills based on self-care to help them develop their psychological and social skills. Life skills training based on self-care improves quality of life and mental health. These trainings may reduce the burden of treatment in the host country and prevent the spread of some diseases. The aim of this study was to evaluate the effectiveness of life skills training program based on self-care on mental health and quality of life of married Afghan women living in Taft.

## Method

### Design

This study was a quasi-experimental study conducted using pretest–posttest design with control group. Statistical population was all married Afghan women living in Taft (Yazd province). Based on the study by Pourmovahed et al. [19] and a preliminary study by the author, standard deviation was 4, significance level was 5% and power of test was 80%. Using purposive sampling, 60 women (Most immigrant women have lived in Iran for 10 years) were selected (Through the health center) and randomly allocated to experimental (n = 30) and control (n = 30) groups. Immigrant women attended the meeting with full consent. There was no obligation to attend the meeting. Inclusion criteria were being married, consent to participate in the study, having self-care ability, absence of speech problems, good fluency and hearing, and having low scores of mental health or quality of life. Exclusion criterion was failing to participate training sessions. After primary evaluations and specifying experimental and control groups, pretest was conducted for both groups. Life skills training program was provided to the experimental group in eight 90-min sessions once a week. The control group received no trainings. At the end of the training program, posttest was conducted on Afghan women (after 2 months). In addition, to observe research ethics, trainings were also provided to the control group in four compressed sessions at the end of the study. Data were analyzed in SPSS 24 using descriptive statistics such as mean and standard deviation and MANCOVA was used to test the research hypothesis. All methods were carried out in accordance with relevant guidelines and regulations. The following questionnaires were used in this study to collect data.

### General Health Questionnaire (GHQ)

The General Health Questionnaire (GHQ) was first developed by Goldberg (1972). This questionnaire aims not to obtain a specific diagnosis of mental illness; rather, it aims to differentiate mental illness and health. In this study, the 28-item questionnaire was used which assesses psychological status of the individual in the last month and includes symptoms such as abnormal thoughts and feelings and aspects of observable behavior here and now. The questionnaire has 4 subscales including physical symptoms, anxiety, insomnia, social dysfunction and depression each of which includes 7 questions. Questions of each subscale are presented in a row; items 1–7 on physical symptoms; 8–14 on anxiety and insomnia; 15–21 on social dysfunction and 22–28 on depression subscale. Likert scale was used for scoring and items are scored as 1, 2, 3 and 4. The total score ranged between 0 and 84. A score of 17 and higher in each subscale and a total score of 41 and higher show deterioration of the participant's condition. Validity of the questionnaire was 0.72 and it was 0.60, 0.68, 0.57 and 0.58 for physical symptoms, anxiety and insomnia, social dysfunction and depression respectively. A *P*-value of < 0.0001 was considered significant [20]. The content validity of this questionnaire for the Afghan men and women population was examined and approved by psychology professors (Psychology faculty members, Yazd university). Due to the cultural and religious similarity of the people of Iran and Afghanistan, no specific case was observed and researchers investigated such cases. In a similar study, the amount of validity was About 0.74 for Afghan population in Iran [6].

### Quality of life of the World Health Organization Questionnaire (WHOQ-BREF)

In order to measure quality of life in this study, the Quality of Life of the World Health Organization Questionnaire (WHOQ-BREF) was used. It consists of 26 items and 4 subscales (physical health, mental health, social relations, and environmental health). The questions were scored on a 5-point Likert scale (Very Negative, Negative, Neutral, Positive, and Very Positive) from 0 to 4.

Reliability of the questionnaire was greater than 0.70 in all domains using Cronbach's alpha. However, in the social relations, Cronbach's alpha was 0.55 which could be due to a small number of questions in this field or its sensitive questions. Validity of the questionnaire was assessed using linear regression with the capability to differentiate between normal and unhealthy groups where there was a significant difference between various fields. Reliability of the above questionnaire was 0.92 using Cronbach's alpha [21]. In this study, the Cronbach's alpha for physical health, mental health, social relations and environmental health was 0.743, 0.798, 0.762 and 0.715 respectively.

### Intervention

The 90-min group training was provided to participants every week. (Taken from the booklet of Shahid Sadoughi University of Medical Sciences, Yazd) It is worth mentioning that participants' adherence to the rules of sessions was also considered. A summary of training sessions is provided in Table 1.

**Table 1:** Content of sessions

Session	Training content
One	Introduction: Familiarity with life skills and self-care, quality of life and mental health behaviors
Two	Self-awareness skills training For self-care,
Three	Communication skills training
Four	Problem solving and decision making training, to deal with self-care barriers
Five	Stress coping training
Six	Physical self-care training
Seven	Spiritual self-care training
Eight	Summing up the previous sessions, submission of comments and suggestions by group members and conducting post-test

## Results

The mean age of participants was 34; the majority of them were illiterate or had education under middle school. In Table 2, the mean and standard deviation of quality of life and mental health scores in the pretest and posttest are presented for experimental and control groups.

**Table 2:** Mean and standard deviation of quality of life and mental health scores for experimental and control groups

Variable	Stage	Group	Mean	Standard deviation
Quality of life	Pretest	Experimental	82.50	6.99
		Control	74.40	7.4
	Posttest	Experimental	89.33	6.46
		Control	83.80	7.09
Mental health	Pretest	Experimental	30.36	9.01
		Control	29.46	11.23
	Posttest	Experimental	35.06	9.24
		Control	30.20	10.16

Results of Table 2 suggested that the mean scores of quality of life in the experimental group following the intervention increased in the posttest (89.33) relative to the pretest (82.50), and the mean scores of mental health also increased in the posttest (35.06) compared to the pretest (30.20).

Results of the Kolmogorov–Smirnov test demonstrated a normal distribution of variables and their dimensions in both groups (*p* > 0.05) and the Levene's test showed that variances were equal (*p* > 0.05). Results of the assuming the equality of the covariance matrix were also approved for quality of life (MBox = 27.848, F = 1.377) and mental health (MBox = 52.604, F = 1.68).

To measure the effect of independent variable on dependent ones, results of multivariate analysis were used which are shown in Table 3.

**Table 3:** Results of multivariate analysis for effectiveness of training program

Independent variable	Tests	Value	F statistic	Significance	Eta squared
Training program	Pillai's trace	0.211	3.617	0.001	0.242
	Wilks' Lambda	0.789	3.617	0.001	0.242
	Hotelling's trace	0.268	3.617	0.001	0.242

Results of Table 3 suggest a significant difference between groups in at least one variable. Results of Lambda's test suggest that the difference between groups is significant at least in one variable (f = 0.789, *p* < 0.001). Given the significant results of multivariate test, results of MANCOVA were used by controlling the effect of pretest to investigate on what variables the training program has had a significant effect. Results are shown in Table 4.

**Table 4:** Results of MANCOVA for the effectiveness of training program in quality of life and mental health

Dependent variable	Source	Total sum of squares	Degree of freedom	Mean squares	F	Significance	Eta squared
Quality of life	Pretest	388.81	1	388.81	9.718	0.003	0.27
	Group	848.8	2	424.4	10.598	0.0001	
Mental health	Pretest	301.465	1	301.46	3.677	0.043	0.130
	Group	696.732	2	348.366	4.24	0.019	

As shown in Table 4, by controlling the pretest effect, the training program had a significant effect on Afghan women's quality of life (*p* < 0.0001) and mental health (*p* < 0.019).

## Discussion

This study was conducted to investigate the effect of life skills training program based on self-care on quality of life and mental health of Afghan women. Results of the study showed that life skills training program based on self-care increased the quality of life and mental health of Afghan women compared to the control group. This finding complies with some studies on the effect of life skills training and its positive effect on quality of life and mental health [16, 17]. Life skills training can strengthen the foundation of the family and prevent the collapse of the family and divorce [22, 23].

Based on the studies by WHO, mental health is defined as a state of well-being in which each individual realizes his/her abilities, can cope with the normal life stresses and is able to help his/her community [24]. In the "Health 2020", WHO states that improving mental health includes people's flexibility in dealing with various life stressors [25]. Life skills enable individuals to have better and more flexible behavior in society and increase their self-esteem [26]. Generally, life skills training has a significant effect on mental health parameters and it may reduce obsessive behaviors in individuals [27]. To explain this finding that life skills training based on self-care has a significant effect on quality of life, it should be noted that life skills training increases individuals' awareness on various aspects of life, and changes their attitude toward life as well as their behavior. This finding complies with the findings of [10, 12, 17]. Paying attention to quality of life components such as attention to body and mind, degree of independence, social relationships, environmental status, spirituality and values in life may increase people's resilience and ability and their adaptation to related problems in life. This will also increase life satisfaction [28]. In the study by Pourmovahed, similar results were obtained on the effect of spirituality on improving mental health and strengthening the foundation of family [29].

## Limitation

This study faced some limitations. First, it was conducted on Afghan women living in Taft and given the cultural differences, care should be taken in generalizing the findings. Another limitation of the study was the limited number of participants and non-random sampling. It is suggested to investigate the effect of life skills training based on self-care on resilience. Variables of this study can be used for both male and female Afghan immigrants as well as Afghan students in other cities with larger sample size. Results of this study can be used in Welfare Organization and counseling and psychological service centers.

## Conclusion

Generally, life skills training based on self-care helps a lot in facilitating adjustment and mental health and prevents the negative effects of stress and even the desire for divorce in couples. Providing the necessary and basic training equips individuals with the set of skills necessary for success in personal life and help them nurture abilities, information, attitudes and skills necessary for a healthy and successful life. Consequently, these factors improve the self-efficacy and quality of life of individuals and strengthen the family, and the individuals use their knowledge, skills and abilities as a resource to protect their health independently [30]. Life skills training based on self-care causes individuals to obtain more knowledge about themselves and the environment surrounding them, find their weak and strong points, become aware of their facilities and limitations, accept reality better and adapt to them more precisely [31, 32]. Life skills training leads to the recognition of rational and irrational thoughts, efficient and inefficient thoughts and positive and negative emotions [33]. Afghan women, considered as minority in the society, may face various problems, can prevent the occurrence of negative psychological characteristics such as stress, anxiety, depression, etc. through learned skills and increase positive psychological characteristics such as self-control, happiness and hope. These trainings allow them to live more comfortably in their social environment by being aware of skills and preventive care. In addition, life skills training is integral to human life and teaching them is a sign of development and advances in the society.

## Data Availability

All data and materials are available. The datasets used or analyzed during the current study available from the corresponding author on reasonable request.
